# Targeted DNA methylation profiling reveals epigenetic signatures in peanut allergy

**DOI:** 10.1172/jci.insight.143058

**Published:** 2021-03-22

**Authors:** Xiaoying Zhou, Xiaorui Han, Shu-Chen Lyu, Bryan Bunning, Laurie Kost, Iris Chang, Shu Cao, Vanitha Sampath, Kari C. Nadeau

**Affiliations:** Sean N. Parker Center for Allergy & Asthma Research at Stanford University and Division of Pulmonary, Allergy, and Critical Care Medicine, Stanford University, Stanford, California, USA.

**Keywords:** Genetics, Immunology, Allergy, Epigenetics

## Abstract

DNA methylation (DNAm) has been shown to play a role in mediating food allergy; however, the mechanism by which it does so is poorly understood. In this study, we used targeted next-generation bisulfite sequencing to evaluate DNAm levels in 125 targeted highly informative genomic regions containing 602 CpG sites on 70 immune-related genes to understand whether DNAm can differentiate peanut allergy (PA) versus nonallergy (NA). We found PA-associated DNAm signatures associated with 12 genes (7 potentially novel to food allergy, 3 associated with Th1/Th2, and 2 associated with innate immunity), as well as DNAm signature combinations with superior diagnostic potential compared with serum peanut–specific IgE for PA versus NA. Furthermore, we found that, following peanut protein stimulation, peripheral blood mononuclear cell (PBMCs) from PA participants showed increased production of cognate cytokines compared with NA participants. The varying responses between PA and NA participants may be associated with the interaction between the modification of DNAm and the interference of environment. Using Euclidean distance analysis, we found that the distances of methylation profile comprising 12 DNAm signatures between PA and NA pairs in monozygotic (MZ) twins were smaller than those in randomly paired genetically unrelated individuals, suggesting that PA-related DNAm signatures may be associated with genetic factors.

## Introduction

Food allergies affect up to 7.6% and 10.8% of children and adults in the United States, respectively (1, 2). Because of the life-threatening potential of anaphylaxis associated with IgE-mediated food allergies, food allergy has become a growing clinical and public health problem (1).

Food allergy is a complex immune disease influenced by an interplay of genetic variants, environmental exposures, gene-environment interactions, and epigenetic modifications (3). Epigenetic modifications have been shown as one of the mechanisms used to adapt to environmental exposures and in mediating gene-environment interactions (4). Epigenetic factors, specifically DNA methylation (DNAm), play important roles in the development of food allergy (3, 5). A study by Canani et al. found higher DNAm levels associated with Th1-related genes (*IL10* and *IFNG*) and lower DNAm levels associated with Th2-related genes (*IL4* and *IL5*) in those with cows’ milk allergy compared with those tolerant to cows’ milk (6). Another study found that peanut oral immunotherapy decreased DNAm of the *FOXP3* gene, a gene associated with tolerogenic responses (7). The study by Martino et al. (8) showed 96 CpG sites that predicted clinical reactivity to food challenge in food-sensitized infants. These sites overlapped with 73 protein-coding genes significantly enriched with the mitogen-activated protein (MAP) kinase canonical pathway. The same group (9) applied genome-wide DNAm to delineate epigenetic modifications in naive T cells activated by bead-bound anti-CD3/anti-CD28 in egg-allergic participants and nonatopic controls, and they found a distinct DNAm profile for genes involved in metabolic and immunological regulation of egg allergy.

These studies are encouraging, and DNAm signatures offer diagnostic and therapeutic potential for food allergy. However, DNAm signatures for food allergy differ between studies, and further research — including exploratory research to identify other potentially novel DNAm signatures — is needed. In this study, we performed targeted next-generation bisulfite sequencing (tNGBS) to evaluate DNAm levels in 125 highly informative genomic regions containing 602 CpG sites for 70 immune-related genes.

We performed tNGBS to evaluate DNAm levels on peripheral blood mononuclear cells (PBMCs) from 10 peanut allergy (PA) and 10 nonallergy (NA) participants, aged 5–10 years old. Among these participants, 5 pairs of participants were (PA versus NA) monozygotic (MZ) twin siblings. The other 10 participants included 5 pairs of dizygotic (DZ) twin siblings (2 pairs PA, 2 pairs NA, 1 pair of PA versus NA). The DZ twin siblings were used as randomly paired genetically unrelated individuals. MZ twins discordant for PA are rare in the population, and the inclusion of these twin samples improves the statistical power by reducing the amount of genetic and/or environmental variability. We observed that the average CpG methylation levels within the targeted genomic regions for 12 genes showed significant differences between PA and NA participants. PA participants also showed decreased DNAm levels at each of 5 CpG sites in the targeted serine protease inhibitor E1 (SERPINE1) region compared with NA participants. Our results indicate PA-associated DNAm signatures at targeted genomic regions are associated with 12 genes, of which 7 are potentially novel to food allergy (brain-derived neurotrophic factor [*BDNF*], *IL17F*, *CXCL12*, *CCR7*, runt-related transcription factor 1 [*RUNX1*], CD3ε, and *SERPINE1*); 3 are associated with Th1/Th2 responses (*IL4*, *IL12B*, and *IL2*) and 2 are associated with innate immune responses (*IL1B* and *IL6*). Using Luminex assay, we found increased secretion of cytokines IL-4, IL-12B, IL-1B, IL-6, CXCL12, and BDNF and secreted protein SERPINE1 following allergen-specific stimulation in PA compared with NA participants. Using stepwise regression analysis and receiver operating characteristic (ROC) curve analysis, 3 combinations of the DNAm signatures from the initial 12 DNAm signatures were selected with top ranked Akaike information criterion (AIC) and AUC. ROC comparison analysis was performed to compare the diagnostic performance of these 3 combinations of DNAm signatures against the existing diagnostic test and serum peanut–specific IgE. Our results indicate that the combinations of DNAm signatures from the initial 12 DNAm signatures had superior diagnostic potential compared with serum peanut–specific IgE for discriminating PA versus NA. We also evaluated the similarity of 12 DNAm signatures between PA and NA participants in MZ twin sibling pairs and randomly paired genetically unrelated individuals using Euclidean distance analysis. We found smaller distances between PA and NA participants in MZ twins compared with randomly paired genetically unrelated individuals, suggesting that the 12 PA-associated DNAm signatures may be associated with genetic factors. Altogether, our results demonstrate 12 food allergy–associated DNAm signatures and differences in protein secretion in response to allergen-specific stimulation between PA and NA participants. In addition, our results indicate the diagnostic potential of DNAm signature combinations for PA and indicate genetic influences on PA-associated DNAm signatures.

## Results

### DNAm signatures were identified in PA.

**Figure 1:**
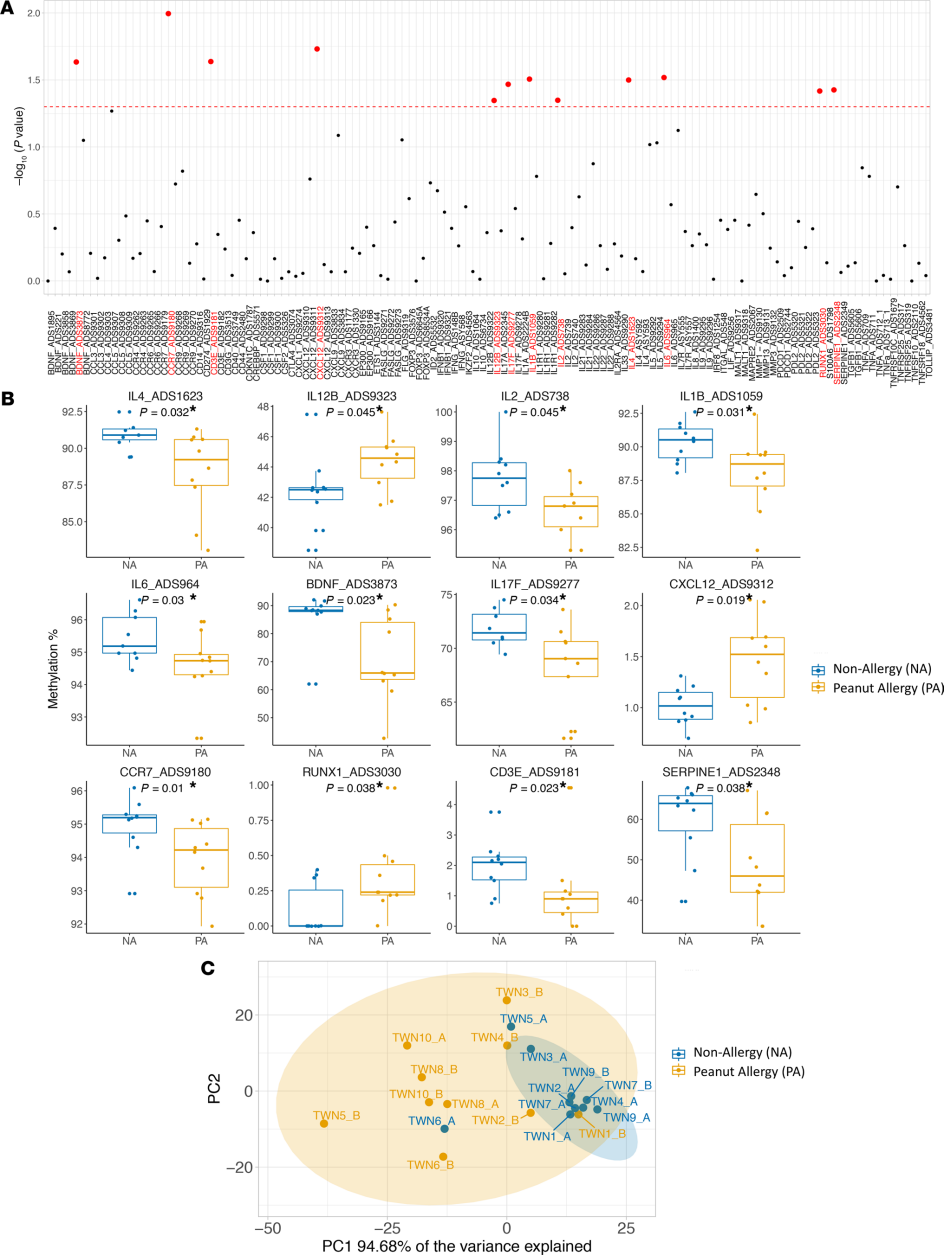
The significant differences in the DNAm levels for 12 targeted genomic regions were observed between PA and NA participants. (**A**) The comparison analysis on the average DNAm level in each of 125 targeted genomic regions between PA (*n* = 10) and NA (*n* = 10) participants was performed using nonparametric unpaired comparison test (Wilcoxon rank sum test). The *y* axis shows the –log_10_ *P* value, and the *x* axis shows 125 targeted genomic regions. The horizontal red dashed line represents a statistical significance level of *P* = 0.05. Each dot represents the *P* value, and the red dots indicate the *P* value less than of 0.05. (**B**) The box plots overlaid with dot plots show the significant differences in the average methylation levels for 12 targeted genomic regions between PA (*n* = 10) and NA (*n* = 10) participants (*P < 0.05). (**C**) PCA of the DNAm levels in the 12 targeted genomic regions shows the 2 distinct clusters formed from NA and PA individuals. The percentage variance explained by principal component (PC) 1 is indicated. Yellow circles represent PA samples; blue circles represent NA samples.

**Figure 2:**
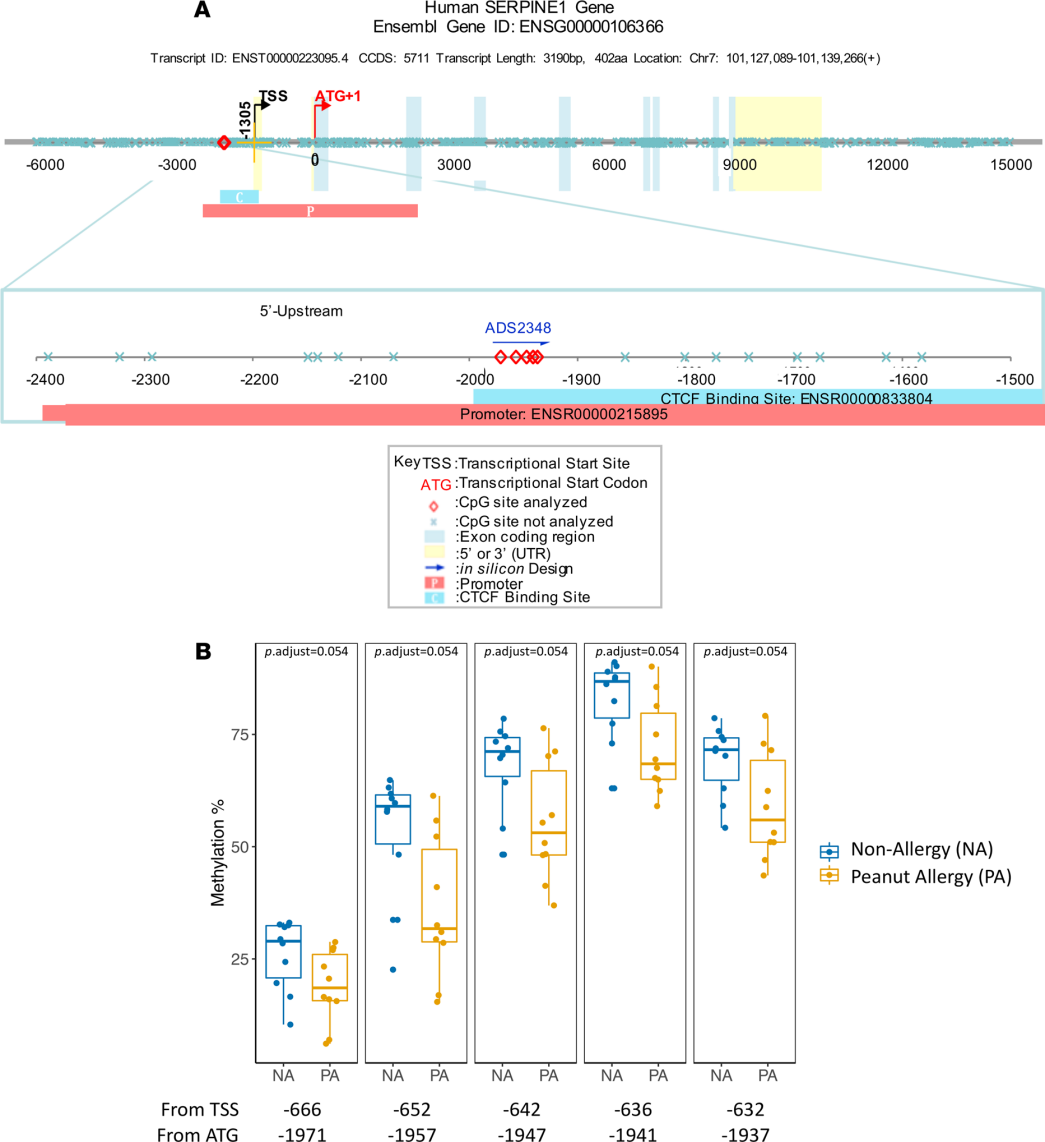
The significant differences in the DNAm levels for 5 CpG sites located in the region of chr7:101126423–101126457 of the *SERPINE1* gene were observed between PA and NA participants. (**A**) Schematic of a genomic region for the SERPIN1 gene with known CpG sites. The 5 CpG sites labeled with red diamond within the targeted genomic region (chr7:101126423–101126457) were analyzed by tNGBS in our study. (**B**) The box plots overlaid with dot plots show the significant differences in methylation levels at each of 5 CpG sites in targeted *SERPINE1* region between PA (*n* = 10) and NA (*n* = 10) participants (adjusted *P* < 0.1). Each dot represents one sample. Box plots indicate the interquartile range (IQR) and median; whiskers extend to the farthest data point within a maximum of 1.5× IQR. Sample sets were analyzed using the Wilcoxon rank sum test (2 sided).

To achieve a set of food allergy–related DNAm signatures, we selected 125 highly informative genomic regions containing 602 CpG sites for 70 immune-related genes based on a comprehensive literature review, DNAm results from our previous study (10), and the genes important immune system function as per existing immunology panel from EpigenDx Inc. (Supplemental Tables 1 and 2; supplemental material available online with this article; https://doi.org/10.1172/jci.insight.143058DS1). tNGBS was performed on PBMCs from 10 NA and 10 PA participants (Table 1). First, we compared the average CpG methylation levels within individual targeted genomic regions using the Wilcoxon rank sum test. Significant differences were observed in the average methylation levels in the 12 targeted genomic regions for 12 genes (*IL4*, *IL12B*, *IL2*, *IL17F*, *IL1B*, *IL6*, *CXCL12*, *BDNF*, *CCR7*, *CD3E*, *RUNX1*, and *SERPINE1*) between NA and PA participants (Figure 1, A and B) (*P* < 0.05; Supplemental Table 3). Next, we compared DNAm levels at each CpG site in the above 12 genomic regions for 12 genes between PA and NA participants (Supplemental Table 3). Of note, the DNAm levels at each of 5 CpG sites in the targeted genomic region (chr7:101126423–101126457) for the gene *SERPINE1* were significantly decreased in PA, compared with NA participants (FDR-adjusted *P* < 0.1) (Figure 2).

The principal component analysis (PCA) of the above 12 DNAm signatures showed that the first principal components accounted for 94.68% of the data set variation and separated PA from NA participants (Figure 1C). To further confirm that the centroid and dispersion of 2 distinct clusters were different between PA and NA samples, we carried out a permutational multivariate analysis of variance (PERMANOVA) for PCA. This test examines the contribution of variables to the separation of the data in multiple dimensional spaces, and it showed a significant *P* value between 2 groups (*P* < 0.001).

We then compared the composition of major immune cell populations between NA and PA participants who are genetically unrelated (demographic characteristics shown in Supplemental Table 4) using flow cytometry and found no differences in major immune cell types, including CD3, CD4, CD8, B cells, NK cells, monocytes, and DCs between NA and PA participants (Supplemental Figure 1). Since there were no statistical differences in the cell phenotypes between groups, it is unlikely that the statistically significant differences in methylation profiles between the groups can be attributed in a major way to cell heterogeneity.

### The secretion levels of the proteins encoded by the genes overlapping DNAm signatures were increased from PBMCs in PA compared with NA participants after peanut stimulation.

**Figure 3:**
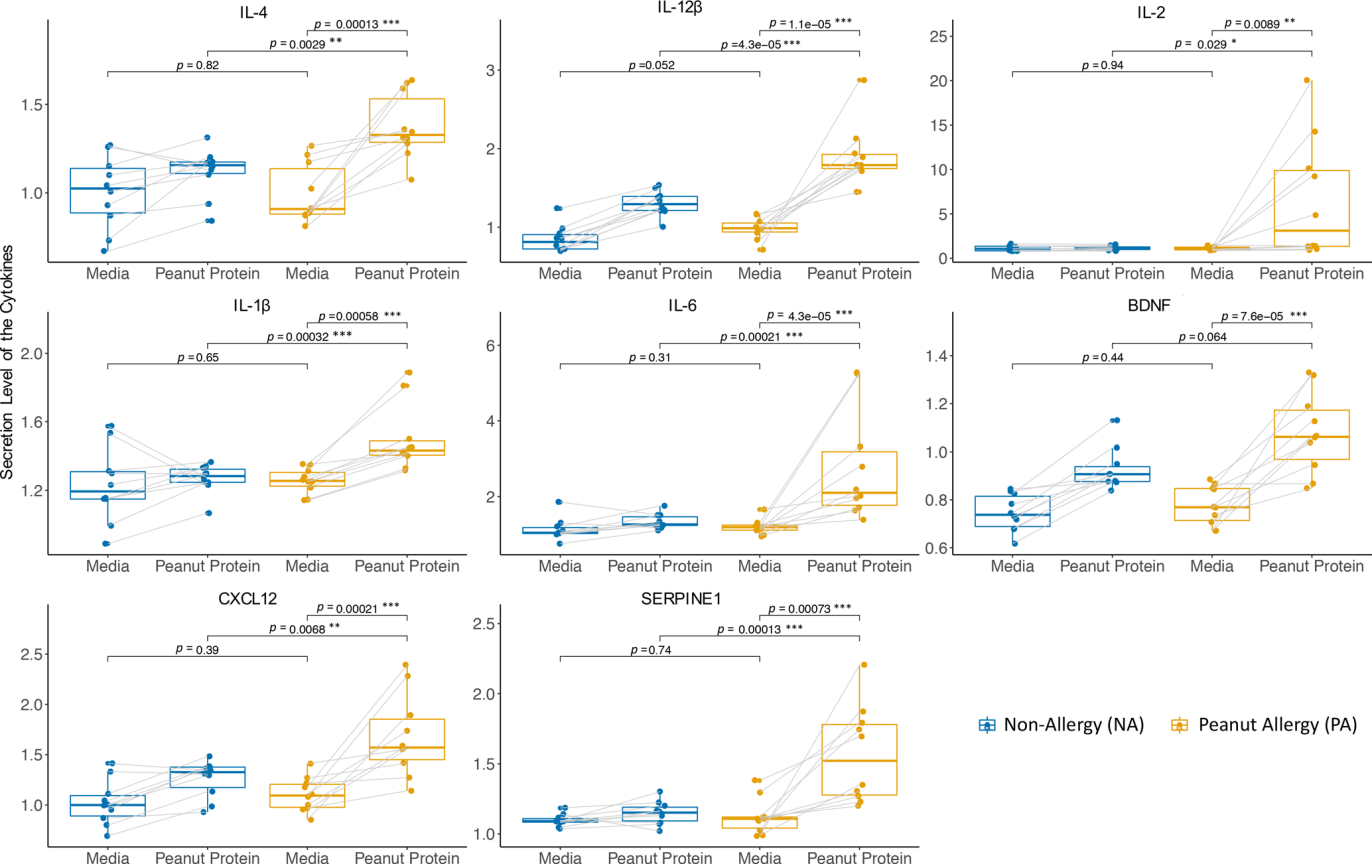
PBMCs from PA participants secrete higher levels of cytokines (IL-4, IL-12β, IL-2, IL-1β, IL-6, CXCL12, and BDNF) and SERPINE1 protein compared with NA participants. Secreted levels of cytokines and SERPINE1 protein from PBMCs stimulated with or without peanut protein for PA (*n* = 10) and NA (*n* = 10) participants are shown in box plots overlaid with dot plots (*P < 0.05, **P < 0.01, ***P < 0.001). Each pair of points connected by a line represents 1 sample. Box plots indicate the IQR and median; whiskers extend to the farthest data point within a maximum of 1.5× IQR. Sets of paired samples were analyzed using the Wilcoxon signed rank test (2 sided). Unpaired sample sets were analyzed using the Wilcoxon rank sum test (2 sided).

Of the above 12 genes showing significant differences in DNAm levels between PA and NA participants, there were 8 cytokine genes (*IL4*, *IL12B*, *IL2*, *IL17F*, *IL1B*, *IL6*, *CXCL12*, and *BDNF*) and 1 gene encoding secreted protein SERPINE1. To determine if these preexisting differential DNAm signatures were associated with expression of their cognate proteins, the PBMCs from NA and PA individuals were incubated either with or without peanut protein. After a 3-day incubation, the secretion levels of these cytokines and SERPINE1 from PBMCs in supernatants were measured using a Luminex-based assay. We found that, compared with NA participants, peanut protein–stimulated PBMCs from PA participants showed increased production of 7 cytokines (IL-4, IL-12β, IL-2, IL-1β, IL-6, CXCL12, and BDNF) and SERPINE1 protein (Figure 3). These results suggest that the differences in protein secretion in response to specific food allergens between PA and NA may be associated with DNAm modifications and environmental interactions.

### The performance of combinations of DNAm signatures for discrimination of PA versus NA participants was superior to serum peanut–specific IgE.

**Figure 4:**
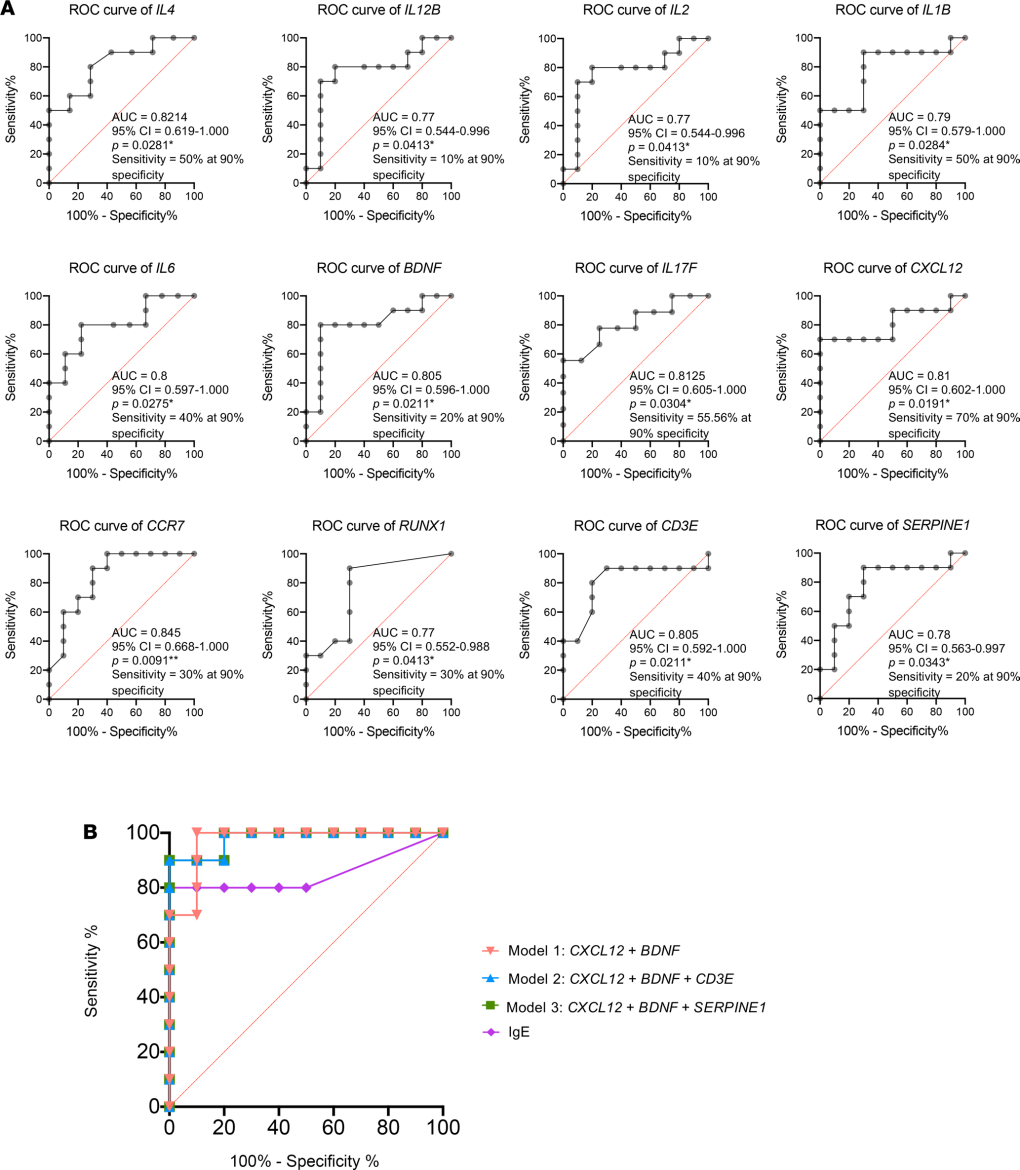
ROC analysis is applied to evaluate the performance of DNAm signatures for discrimination of PA versus NA groups. (**A**) ROC curves show the AUC and the sensitivity and specificity for each of the 12 DNAm signatures. (**B**) ROC curves show the AUC, and the sensitivity and specificity, for comparison of 3 models with the combination DNAm signatures against peanut-specific IgE in serum.

Next, we performed ROC analysis to calculate the AUC of 12 DNAm signatures. The AUC for 12 individual ROC curves varied from 0.77 to 0.845 for PA versus NA. Diagnostic sensitivity for individual DNAm signatures at 90 % specificity ranged from 10% to 70 % for PA versus NA (Figure 4A). On the basis of the assumption that each DNAm signature can be considered as a diagnostic test, we attempted to optimize DNAm signature combinations from the initial 12 DNAm signatures. We applied a stepwise (step-up) regression analysis and selected 3 models that have top-ranked AIC and AUC. The β value (standardized regression coefficients) of the 3 models are as follow: model 1, +82.518 (*CXCL12*) – 2.5 (*BDNF*) + 107.287; model 2, +134.199 (*CXCL12*) – 2.230 (*BDNF*) + 12.416 (*CD3E*) – 10.798; model 3, +73.62 (*CXCL12*) – 1.543 (*BDNF*) – 1.267 (*SERPINE1*) + 85.039 (Table 2). To determine whether these 3 models with the combination of DNAm signatures could exceed the diagnostic potential of existing diagnostic tests, we compared the above ROC curves against peanut-specific IgE levels in serum (Figure 4B). The 3 models with the combination of the DNAm signatures had the AUC of 0.97 for model 1 (*CXCL12* + *BDNF*), 0.98 for model 2 (*CXCL12* + *BDNF* + *CD3E*), and 0.98 for model 3 (*CXCL12* + *BDNF* + *SERPINE1*), respectively, compared with 0.85 for serum peanut–specific IgE (Figure 4B and Table 2). The sensitivities at 90 % specificity for discrimination of PA versus NA for 3 models with the combination of the selected DNAm signatures were 70% (model 1), 90% (model 2), and 90% (model 3), respectively. These results suggest that DNAm signatures can be combined to produce highly clinically sensitive and specific DNAm panels, and the above 3 combinations of DNAm signatures have superior diagnostic potential compared with peanut-specific IgE in serum (Figure 4B and Table 2).

### PA-associated DNAm signatures were genetically influenced.

**Figure 5:**
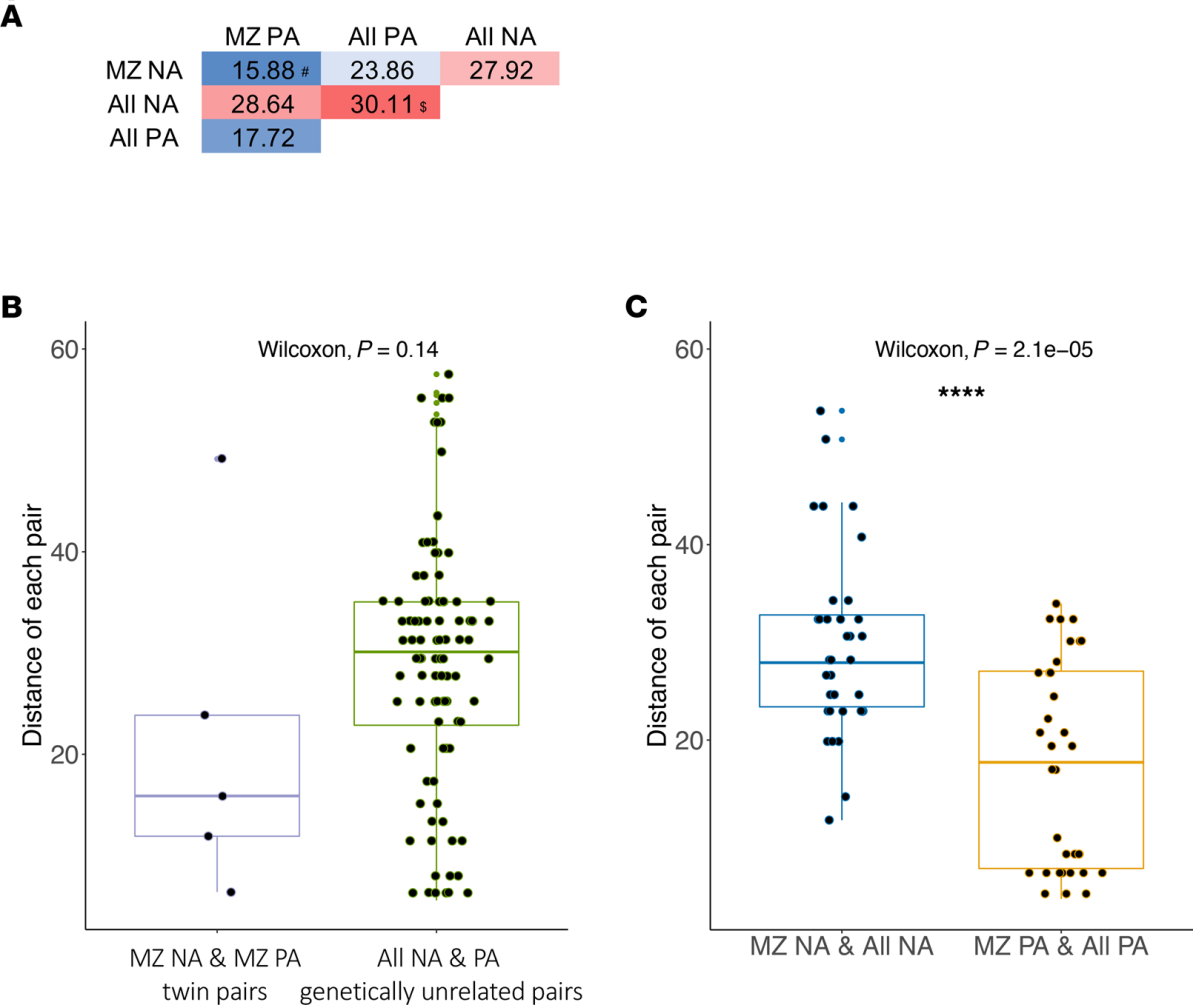
Peanut allergy–associated DNAm levels in 12 targeted genomic regions are genetically influenced. (**A**) Euclidean distances of 12 DNAm signatures were calculated pairwise either between 5 MZ twin pairs (5 pairs) who are discordant for peanut allergy or randomly selected genetically unrelated pairs (95 pairs) (i.e., 1 sample has peanut allergy and the other is nonallergy without peanut allergy). ^#^Median of the distance within 5 discordant MZ twin pairs. ^$^Median of the distance of all 95 genetically unrelated NA and PA pairs. (**B**) Box plots overlaid with dot plots represent the Euclidean distances between MZ PA and NA twin pairs (pairs = 5, left panel) and genetically unrelated individuals in PA participants and in NA individuals (pairs = 95, right panel). (**C**) Box plots overlaid with dot plots represent the Euclidean distances between random pairs, of which 1 of each pair is an NA MZ twin and the other is a genetically unrelated NA individual (pairs = 35, left panel), and random pairs, of which 1 of each pair is a PA MZ twin and the other is a genetically unrelated PA individual (pairs = 35, right panel). Box plots indicate the interquartile range (IQR) and median; whiskers extend to the farthest data point within a maximum of 1.5× IQR. The Wilcoxon rank sum test (2 sided) was used for comparison analysis. ***P < 0.001.

Among the 10 PA and 10 NA participants, 5 pairs were PA discordant MZ twin siblings aged 5–10 years old (Table 1). Since MZ twin pairs are widely regarded as genetically identical, and young twins usually share similar environmental backgrounds, the discordant PA in MZ twin pairs are presumed to result from different epigenetic mechanisms. The epigenetic drift during the lifetime of MZ twin pairs has been suggested to arise from differing environmental histories (11, 12). In contrast, the contributions of both environmental in utero and underlying genetic factors to epigenetic profile have been suggested in neonatal epigenome for MZ twins with discordant phenotypes (13). To estimate relative contributions of the genetic factors to the epigenetic profile comprising 12 DNAm signatures, we performed a Euclidean distance-based analysis to compare the similarity of food allergy–associated epigenetic profile in the MZ twins and the randomly paired genetically unrelated individuals. The median of distances between PA and NA pairs in the MZ twins decreased by 48.28% compared with the median distances in the randomly paired genetically unrelated individuals, although the results did not reach statistical significance (*P* = 0.14) (Figure 5, A and B). The smaller distance between PA and NA participants in MZ twins suggests that PA-related DNAm signatures may be associated with genetic factors. Further studies are needed to test if this is significant. We also observed a smaller distance between MZ twins and genetically unrelated individuals in PA participants compared with NA participants; this suggests epigenetic similarities for the above 12 DNAm signatures in PA, but not NA, regardless of whether the PA participants were MZ twin siblings or genetically unrelated individuals (Figure 5, A and C).

## Discussion

This study presents potentially novel findings on the loci differentially methylated for food allergy. The PA-associated DNAm signatures at the targeted genomic regions for 12 genes were identified by comparing the DNAm levels in 125 targeted genomic regions containing 602 CpG sites for 70 immune-related genes between PA and NA participants. These DNAm signatures for 12 genes include the genes associated with Th1/Th2 differentiation (*IL4,* *IL12B*, and *IL2*), innate immunity (*IL1B*, *IL6*), and those involved with immune regulation but not specifically for food allergy, such as *BDNF*, *IL17F*, *CXCL12*, *CCR7*, *RUNX1*, *CD3E*, and *SERPINE1*. Incubating PBMCs from PA participants with peanut protein resulted in the increased secretion of IL-4, IL-12β, IL-2, IL-1β, IL-6, CXCL12, BDNF, and SERPINE1, compared with NA participants, which may suggest that the differences in PBMC responses on stimulation with specific food allergens between PA and NA groups are associated with DNAm changes and environmental interactions. In addition, our data show that 3 combinations of DNAm signatures from the 12 PA-associated DNAm signatures have superior diagnostic performance against serum peanut–specific IgE for discriminating PA versus NA. Our results also demonstrate that PA-associated 12 DNAm signatures are influenced by genetic factors.

It has long been understood that IgE-mediated food allergy results from a Th2 immune response of the adaptive immune system to protein antigens associated with specific foods (14). Therefore, the skewing of naive CD4^+^ T cell differentiation into Th1 or Th2 effector cells, driven by the cytokine environment, is critical to the development of food allergy. Cytokine IL-12 is mainly produced by phagocytic cells (monocytes, macrophages, neutrophils and DCs) and has been shown to drive naive T cells to differentiate into Th1 cells. IL-4 (produced by Th2 cells) is a major cytokine driving the differentiation of naive T cells into a Th2 subset (15). In addition, studies have suggested that IL-2 also has a role in facilitating Th2 differentiation (16). Low doses of IL-2–induced Treg expansion provides protection against clinical manifestation of food allergy by Treg-dependent modification of Th1/Th2 balance (17). Consistent with previous studies showing the epigenetic regulation of Th1/Th2 differentiation (18), compared with NA participants, PA participants showed a decrease in DNAm levels at the targeted genomic regions of *IL4* and *IL2*, as well as an increase in DNAm levels at the targeted genomic region of *IL12B*.

The proinflammatory cytokines IL-1β and IL-6, mainly produced by different innate immune cells, were significantly increased in food-allergic compared with NA participants when stimulated by LPS (19) or by specific allergens, as shown in Figure 2. These observations suggest that different innate immune responses occur in food-allergic versus nonallergic participants. A study demonstrated that exposure to LPS (tolerance immunity) or bacterial β-glucan (trained immunity) induces epigenetic changes in monocytes. These reprogrammed epigenetic landscapes of innate immune cells determine the capacity for developing a “memory” in response to exogenous exposure (20). In our study, we also observed significant decreases in DNAm levels for the gene *IL1B* and *IL6* in PA compared with NA individuals, suggesting a link between epigenetic regulation of the innate immune system and food allergy.

It is becoming increasingly clear that immune cells do not act alone and that crosstalk and reciprocal regulation between neural and immune systems are essential in the pathophysiology of allergic diseases, including allergic asthma, atopic dermatitis, and food allergies (21, 22). Both immune and neural cells detect and respond to environmental threats and harmful stimuli, including allergens. Proinflammatory mediators, such as cytokines or chemokines, mediate allergic responses and also directly activate sensory neurons that regulate itch, sneezing, bronchoconstriction, and alterations in gastrointestinal motility (23). However, the mediators between neuronal and immune cells and their role in mediating allergic responses remain unclear. BDNF is a member of the neurotrophin family, which is known to be related to canonical nerve growth factor and neurogenic inflammation (24, 25). Recently, neurotrophins were also found to be produced continuously during allergic inflammation (26). Increased expression of BDNF has been observed in severe asthma, bronchial hyperresponsiveness, and inflammation (26), but to our knowledge, it has not been observed in food allergy. Our results, show that DNAm levels of *BDNF* are significantly decreased in PA compared with NA participants, suggesting that epigenetic modification of the BDNF gene is associated with food allergy. In addition, the combinations of DNAm signatures of *BDNF* and *CXCL12* had the most superior diagnostic potential among all other combinations selected compared with serum peanut–specific IgE for discriminating PA versus NA.

Of note, the average DNAm level in the targeted *SERPINE1* genomic region (chr7:101126423–101126457) and the DNAm levels at each of 5 CpG sites in this targeted region were significantly decreased in PA compared with NA participants. In mice, following intranasal OVA challenge, WT mice skewed to a Th2 immune response while *SERPINE1*^–/–^mice skewed from a Th2 to a Th1 immune response (27). In house dust mite (HDM) allergic asthma patients, SNP (rs1799768) in the *SERPINE1* gene was associated with bronchial reactivity to histamine and IgE response (28). Interestingly, the gene *SERPINE1* 5′-upstream promoter region (chr7:101126423–101126457; Figure 1C) contains the allergic disease–associated SNP (rs1799768, chr7:101126426) (28). It has been proposed that epigenetic modification may mediate the effect of genetic variants on the development of food allergy (3). For example, the DNAm of HLA-DQB1 and HLA-DRB1 genes were implicated in the mediation of the association between SNPs in HLA-DQ and PA and between HLA-DR and PA (29). Consistent with this possibility, the observation on the genomic location of allergy-associated *SERPINE1* SNP suggests that the DNAm in the above targeted regions for the SERPINE1 gene might act as a mediator of the association between genetic variation and allergic disease.

IL-17F and IL-17A are related homodimeric proteins of the IL-17 family and are produced by Th17 cells. Both 17F and IL-17A cytokines have been implicated in allergic inflammation (21) and inflammation resulting from mucosal immunity or autoimmunity (30). It has been suggested that IL-17A plays a role in the regulation of food allergy and is a potential biomarker of tolerance to food allergens (31); however, the role of IL-17F in food allergy is not well understood. Our results suggest that epigenetic modification of the *IL17F* gene is associated with food allergy.

Increased levels of the chemokine CXCL12 has been found in the bronchoalveolar lavage of patients with asthma (32). Overexpression of the chemokine receptor CCR7 in DC is thought to mediate DC lymph node migration and promote the development of allergic responses (33). Genome-wide association studies (GWAS) show that the SNPs in *RUNX1* are associated with airway responsiveness in asthmatic children (34), and the RUNX1 transcription factor has been identified as a molecular link in TGF-β–induced FOXP3 expression in inducible Treg (35). It has been proposed that the signals transduced by CD3ε contribute quantitatively to TCR signaling and that CD3ε signals suggest a potential role in the generation and/or survival of mature T cells (36). Here, our results show the association between DNAm levels with food allergy for *CXCL12*, *CCR7*, *RUNX1*, and *CD3E*.

DNAm is most commonly associated with downregulation of gene expression — especially when the hypermethylation is in the promotor region of a gene. In our study, *IL4*, *IL2*, *IL1B*, *IL6*, *BDNF*, and *SERPINE1* showed lower methylation level and higher protein level in the PA compared with NA participants. However, the PA patients have increased DNAm at 5′ upstream of *IL12B* and *CXCL12*, which is associated with increased IL-12β and CXCL12 protein expression compared with NA participants. It should be noted that, although gene silencing by promoter hypermethylation seems to be the most likely mode of action, there is growing evidence of a more complex view on the effect of DNAm in various contexts (37–41). In particular, for genes that become methylated, the associated expression level can be unaffected or even upregulated in some cases (40), suggesting a more diverse mechanism of epigenetic regulation. Such additional complexity could have important implications for understanding allergic disease but has not been studied at a genome-wide scale.

Among the 10 PA and 10 NA participants in this study, 7 are allergic to tree nuts, 4 have asthma, and 6 have atopic dermatitis. To examine whether similar DNAm signatures are presented in other allergic disease, we applied the Wilcoxon rank sum test to compare the average CpG methylation levels within individual targeted genomic regions using our original data (total 125 targeted genomic regions covering 602 CpG sites) between 6 atopic dermatitis versus 14 nondermatitis participants, 4 asthma versus 16 nonasthma participants, and 7 tree nut–allergic versus 13 non–tree nut–allergic participants. The differential methylated genomic regions related to atopic dermatitis, asthma, or tree nut allergy are presented in Supplemental Figures 2–4. Among the 10 differentially methylated genomic regions associated with atopic dermatitis (Supplemental Figure 2), none of them overlap with the 12 PA-associated differentially methylated genomic regions, and all of the 10 genes have been reported to be associated with atopic dermatitis in previous studies (42–51). Of note, the differential DNAm in the genomic region of the filaggrin gene (*FLG*), which plays an important role in the pathogenesis of atopic dermatitis and allergic disease (42, 52), is identified in our comparison analysis between nonatopic dermatitis and atopic dermatitis participants. Among 4 differentially methylated genomic regions associated with asthma (Supplemental Figure 3), 2 of them (*IL12B*, *IL2*) overlap with the 12 PA-associated differentially methylated genomic regions, and all of the 4 genes have been reported to be associated with asthma in previous studies (53–57). Among 9 differentially methylated genomic regions associated with tree nut allergy (Supplemental Figure 4), only 1 (*CXCL12*) overlaps with the 12 PA-associated differential methylated genomic regions, and 6 of the 9 genes (*IL10*, *IKZF2*, *CCL5*, *IL21*, *CCR9*, and *IL-33*) have been reported to be associated with food allergy in previous studies (58–63). All of the above results support that the 12 differentially methylated genomic regions are, to some extent, specific to PA.

Oral food challenges (OFCs) are the gold standard for diagnosis of food allergy; however, OFCs are associated with risk of allergic reaction and, therefore, need to be performed in clinics with trained staff; this practice limits widespread use (64). The study by Martino and colleagues (8) showed that DNAm signatures at 96 CpG sites can predict food challenge outcomes by comparing the differences of DNAm between food allergic and food sensitization groups. Their results show that the 73 genes overlapped by these 96 CpG sites were enriched in the sole MAP kinase canonical pathway but were not involved in the well-known food allergy–associated Th1/Th2 pathway. In addition, these clinically relevant biomarkers have not been widely used in clinic diagnosis. We also compared the diagnostic performance for these 12 DNAm signatures for PA against peanut-specific IgE in serum. We found that diagnostic sensitivity for peanut-specific IgE at 90% specificity is 80%, but the sensitivity of individual DNAm signatures at 90% specificity ranged from 10% to 70 %. To select the optimal combination of DNAm signatures from the initial 12 DNAm signatures, we further performed stepwise regression analysis and ROC curve analysis, and our pilot exploratory results indicate that the combinations of DNAm signatures selected from the initial 12 DNAm signatures had superior diagnostic potential compared with serum peanut–specific IgE for discriminating PA versus NA.

The birth rate for MZ twins is about 0.3% of the world population, and a previous study showed that, among 14 pairs of MZ twins, only 5 of them were discordant for PA (65). The young allergy-discordant MZ twin participants used in this study have nearly perfect controls of covariates such as age, sex, and genetic and environmental factors, which increase the rigor and reproducibility of our epigenetic association studies. It increases the power estimation over ordinary case-control designs by minimizing confounding genetic and environmental factors (66, 67). In addition, to avoid reporting findings caused by natural variability within twin pairs, we compared our data with a study that investigated genome-wide DNAm variability in adolescent MZ twins followed since birth (68). This study showed that probes with the highest within-pair differences in DNAm were enriched in gene ontologies related to development and cell growth. The PA-associated 12 differential methylated gene regions identified in our study do not overlap with the hypervariable genes across MZ twins in previous studies (68). The lack of overlap between the 2 studies, in gene variability within twin pair differences in DNAm, reduces the likelihood that our findings were due to general within-pair variability and increases our confidence that these data represent true methylation difference associated with PA. The development of these combinations of DNAm signatures for diagnosis of PA need further verification in detailed follow-up studies with large sample size.

Overall, our results reveal that the food allergy–associated DNAm signatures suggest epigenetic modifications could discern PA versus NA individuals, and that these DNAm signatures could be potentially used for diagnostics or future medical research in not only PA, but also food allergy. Several DNAm signatures highlighted in the current study have been associated with PA, suggesting that these genes may have important roles in food allergy with potential as biomarkers and potential for targeted therapy. Further validation with a larger cohort and further functional studies are warranted to enable further understanding of the molecular mechanisms underpinning food allergy. The potentially novel PA-associated DNAm genes, such as BDNF (neurotrophin) and SERPINE1 (serine protease inhibitor), suggest an additional direction of research for deciphering the molecular basis of PA. Furthermore, a high similarity between PA and NA participants was observed in MZ twins compared with randomly paired genetically unrelated individuals, indicating that the PA-associated 12 DNAm signatures were genetically influenced.

## Methods

### Study participants.

PA discordant MZ twin siblings, nontwin PA pediatric participants, and nontwin NA pediatric participants were recruited at the Sean N. Parker Center for Allergy and Asthma Research at Stanford University. Patient demographics, food allergy history, atopic history, and peanut-specific IgE are summarized in Table 1. PA was confirmed by a food challenge by a certified allergy specialist. Blood specimens were drawn before food challenges were performed, and no participant was taking any medications (e.g., steroids, valproic acid, folic acid, DNA intercalating agents, methotrexate, or DNA methyltransferase inhibitors) that could have affected the epigenetics.

### Collection and processing of blood specimens.

PBMCs and plasma were isolated from blood samples by density gradient centrifugation (400g for 20 minutes at room temperature) over Ficoll-Paque. PBMCs were cryopreserved in 10% dimethyl sulfoxide in FCS and stored in liquid nitrogen. Plasma was stored at –80°C.

### tNGBS.

tNGBS was performed on PBMCs from 10 PA and 10 NA participants by EpigenDx Inc. In total, 125 targeted genomic regions containing 602 CpG sites for 70 genes were analyzed using tNGBS. Targeted bisulfite deep-sequencing PCR products were purified using QIAquick PCR purification kit (QIAGEN). Ion Torrent deep-sequencing libraries were constructed from bisulfite-converted DNA using the KAPA Library Preparation Kit (Kapa Biosystems), quantified using the QIAxcel Advanced System (QIAGEN), templated using the Ion PGM Template OT2 200 kit (Thermo Fisher Scientific), and sequenced using the Ion PGM Sequencing Hi-Q OT2 Kit with the Ion 314 Chip Kit v2 on an Ion PGM System (Thermo Fisher Scientific), which generated nondirectional, approximately 200 nt–length reads at 1500–7500 reads per library in the fastq format. FASTQ files from the Ion PGM System were filtered and aligned to the human genome assembly hg38 using Bismark Bisulfite Mapper v0.15.0 (Babraham Bioinformatics) with the Bowtie 2 alignment algorithm. Methylation levels were calculated in Bismark by dividing the number of cytosine converted (cytosine versus thymine; C versus T) reads by the number of total reads, considering all CpG sites covered by a minimum of 30 total reads. The DNAm level at each of 602 CpG sites were reported by EpigenDx.

### In vitro stimulation.

The peanut proteins added to cell culture were derived from peanut flour used for double-blind placebo–controlled food challenges (DBPCFCs) in the clinic. The peanut flour was dissolved in PBS and sterilized by filtration. The peanut protein concentration was determined by BCA Protein Assay (Pierce). The endotoxin level of peanut protein was assessed by fluorescence-based rFC assay (Indoor Biotechnologies), and the endotoxin level of peanut protein exhibited in cell culture was 0.05 EU/mL.

After overnight resting of thawed PBMCs, cells were cultured in complete RPMI medium (RPMI1640 medium [Thermo Fisher Scientific], glutamine [Thermo Fisher Scientific], 5% human serum [MilliporeSigma], 1% penicillin/streptomycin [Thermo Fisher Scientific]) and in the presence or absence of peanut protein at a final concentration of 100 μg/mL for 3 days. For each condition of this experiment, PBMCs were cultured at 5 × 10^5^ cells per 200 μL for 3 days, after which the supernatants were harvested and stored at –80°C.

### Cytokine assays.

The secretion levels of cytokines or chemokines from PBMCs in supernatants were measured using a 62-multiplex assay on the Luminex 200 IS system (Affymetrix) performed by Stanford Human Immune Monitoring Center (HIMC). All samples were tested in duplicate wells. Data were analyzed using MasterPlex software (Hitachi Software Engineering America Ltd., MiraiBio Group), and the average of 2 median fluorescence intensity (MFI) values for each sample for each analyte were reported by Stanford HIMC. Then, the ratios were calculated by dividing the average MFI of each analyte for each sample by the average MFI of each analyte for complete RPMI medium control. These ratios were used to present the secretion level of each cytokine or chemokine from PBMCs for each sample.

### Flow cytometry.

PBMCs from 10 NA and 10 PA participants were stained with the lineage markers listed in Supplemental Table 5 to examine the proportion of major immune cell subtypes in total PBMCs. The PBMCs (about 1 × 10^6^ cells per sample) were incubated in 100 μL volume with Human TruStain FcX (BioLegend), cell surface antibodies (in Supplemental Table 5) and viability dyes (Aqua, Thermo Fisher Scientific) for 30 minutes on ice, and followed by washing 2 times with FACS buffer (PBS with 0.25% BSA and 1 mM EDTA). All samples were run on LSRII flow cytometer and analyzed using FlowJo Version 10.6.0 software.

### Statistics.

The analysis was conducted using the statistical programming language R (version R 3.6.2). The differences in average methylation across all CpGs in each targeted genomic region and the differences in DNAm level at each CpG site in individual targeted genomic region, were compared between PA and NA participants using Wilcoxon rank sum test (nonparametric unpaired test, 2 sided). A *P* value less than 0.05 was considered significant. Using the Benjamini-Hochberg procedure with an FDR of 0.1, the assay-wise multiple testing correction was further performed across each of the CpG sites in individual targeted genomic region (or individual independent assay). An adjusted *P* value less than 0.1 was considered significant.

The mixed-effects logistic regression correcting for the dependent participants, such as MZ twin samples, was conducted using the “glmer” function in the “lme4” R package (version 1.1-23). The DNAm signatures used for regression and creating the ROC curve are the average methylation levels across the CpG sites within a targeted genomic region, which were detected by tNGBS by 1 pair of primers. The step-up regression was performed to select the combination of variables in terms of the AIC value and AUC value. Three models with the combinations of 2 or 3 methylation signatures have the both top 3 lowest AIC values and top 3 highest AUC values. Equivalent ROC curves were obtained from mixed-effects logistic regression. ROC analysis was performed using Prism 8 (GraphPad).

PCA was then conducted to visualize the DNAm signatures between NA and PA groups using pcaMethods package in R. PERMANOVA was applied to examine the contribution of variables to the separation of the data in multiple dimensional space using the “adonis2” function in the “vegan” package in R. The distance function in R software was employed for Euclidean distances analysis. All dot plots overlaid with boxplots or line connections were compiled with ggplot2 package in R.

### Study approval.

The study was approved by the IRB of Stanford University and registered at Clinicaltrials.gov (NCT01613885). All participants or their caregivers provided written informed consent.

## Author contributions

KCN conceived the study. XZ and SCL performed experiments and collected data. BB and LK contributed to the recruitment of study participants. IC contributed to processed samples for this study. XZ, XH, SCL, and KCN contributed to experimental design, data analysis, and data interpretation. SC assisted with statistical analyses. XZ, XH, VS, and KCN wrote the paper. XZ and XH contributed equally to this research as co–first authors. The order of appearance of the co–first authors was based on the timeline of contributions to the work.

## Supplementary Material

Supplemental Tables 1-5

Supplemental data

## Figures and Tables

**Table 1:**
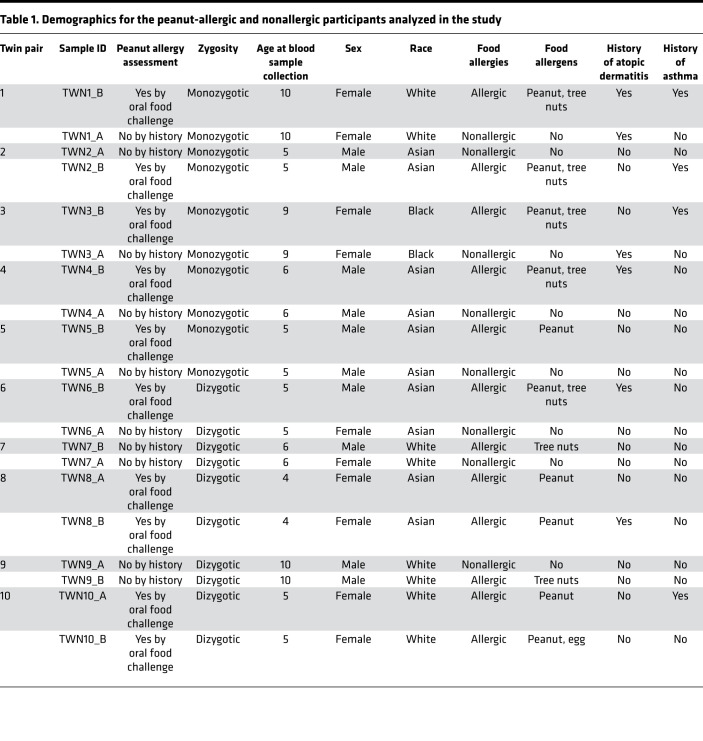
Demographics for the peanut-allergic and nonallergic participants analyzed in the study

**Table 2:**
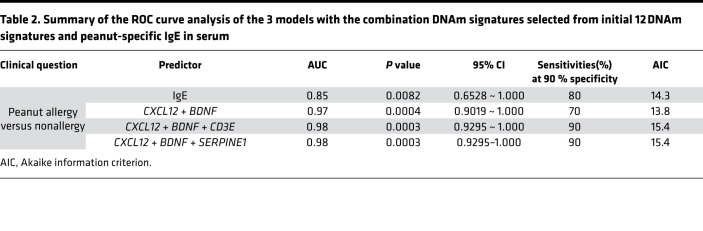
Summary of the ROC curve analysis of the 3 models with the combination DNAm signatures selected from initial 12 DNAm signatures and peanut-specific IgE in serum

## Version 1 02/11/2021

In-Press Preview

## Version 2 03/22/2021

Electronic publication
