# Supplementary material for: Targeted DNA methylation profiling reveals epigenetic signatures in peanut allergy
Source: JCI Insight. 2021 Mar 22;6(6):e143058. doi: 10.1172/jci.insight.143058 (PMC8026193; doi:10.1172/jci.insight.143058)
Supplement: Supplemental data [file jciinsight-6-143058-s052.pdf]

Supplementary Figure 1

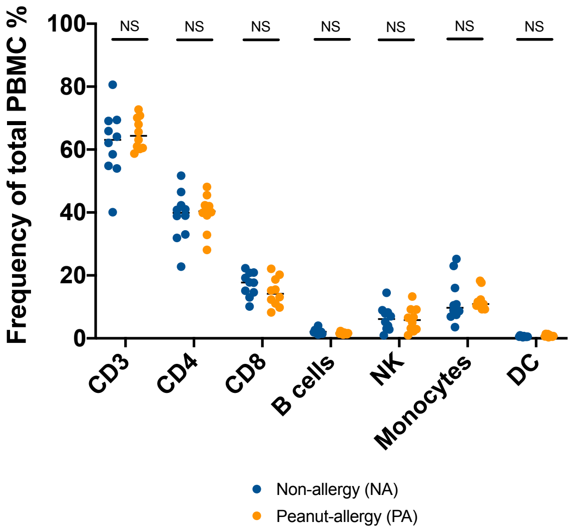

Supplementary Figure 1. There are no differences in the frequency of main immune cell types between non-allergic and peanut allergic subjects. Wilcoxon rank sum tests were used for unpaired comparisons.

Supplementary Figure 2

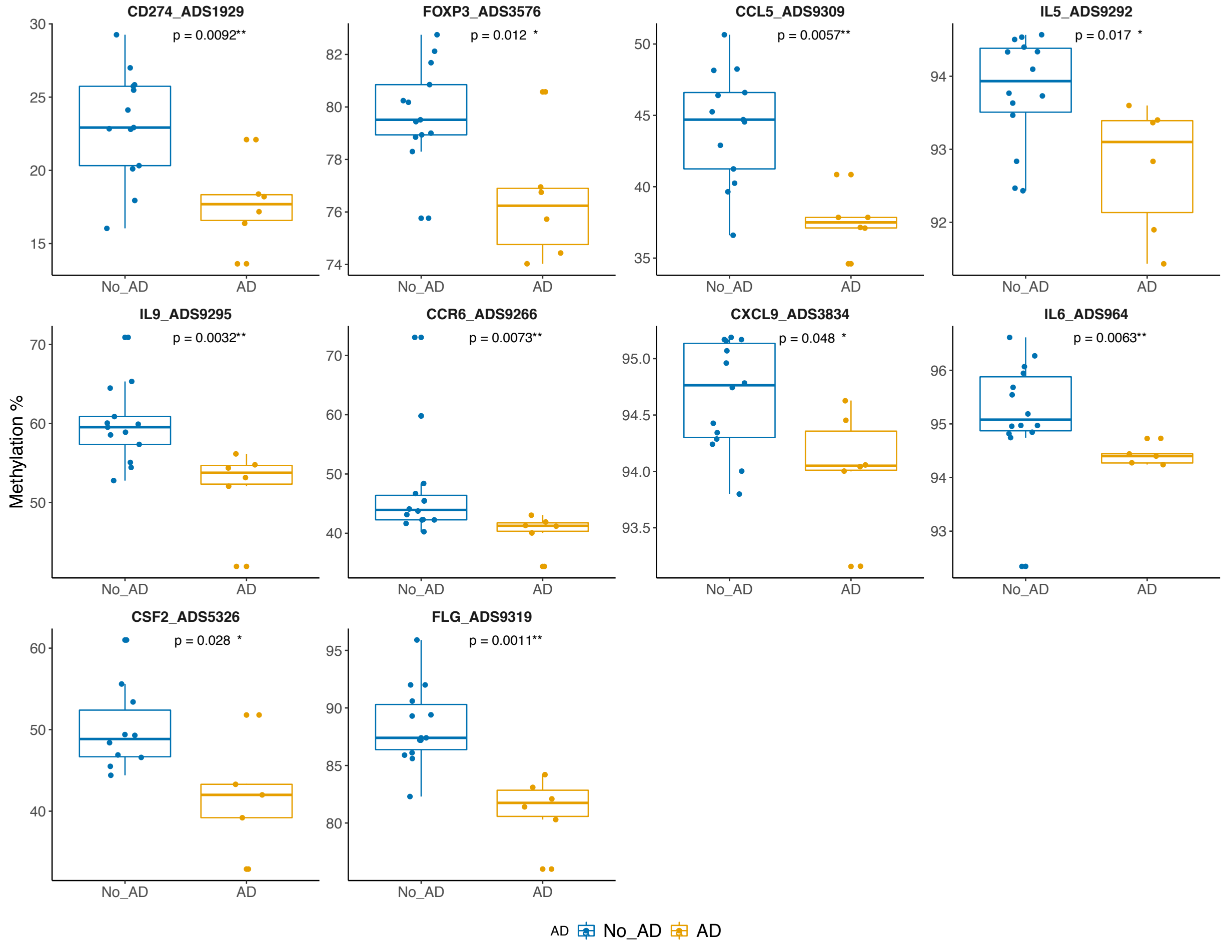

Supplementary Figure 2. The significant differences in the DNAm levels for targeted 10 genomic regions were observed between no atopic dermatitis (n=14) and atopic dermatitis (n=6) participants. The comparison analysis on the average DNAm level in each of 125 targeted genomic regions between no atopic dermatitis (n=14) and atopic dermatitis (n=6) participants was performed using nonparametric unpaired comparison test (Wilcoxon rank sum test). The box plots overlaid with dot plots showed the significant differences in the average methylation levels for 9 targeted genomic regions between no atopic dermatitis (n=14) and atopic dermatitis (n=6) participants (*p<0.05, **p<0.01).

Supplementary Figure 3

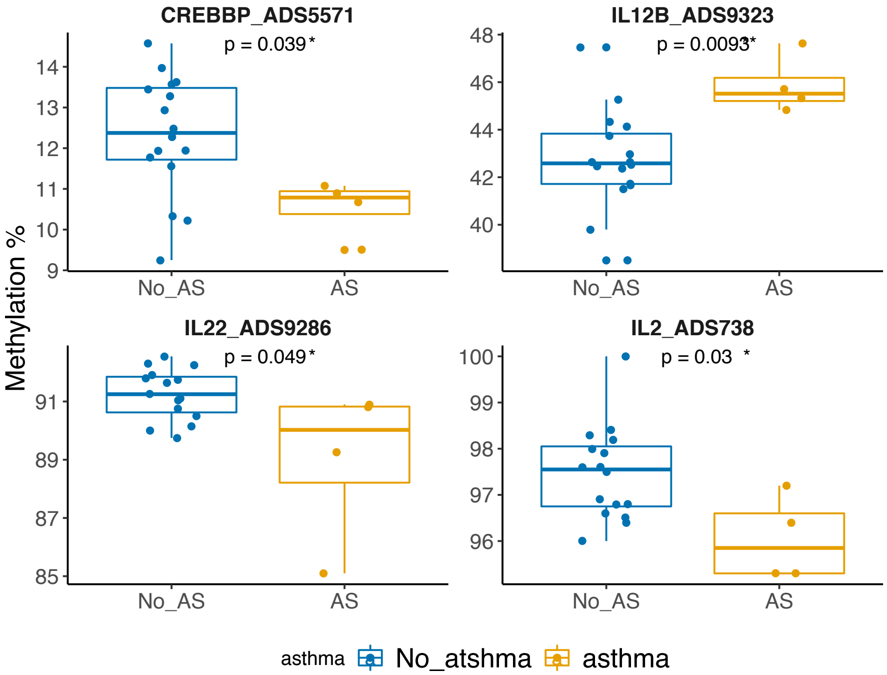

Supplementary Figure 3. The significant differences in the DNAm levels for targeted 4 genomic regions were observed between no asthma (n=16) and asthma (n=4) participants. The comparison analysis on the average DNAm level in each of 125 targeted genomic regions between no asthma (n=16) and asthma (n=4) participants was performed using nonparametric unpaired comparison test (Wilcoxon rank sum test). The box plots overlaid with dot plots showed the significant differences in the average methylation levels for 9 targeted genomic regions between no asthma (n=16) and asthma (n=4) participants (*p<0.05)

Supplementary Figure 4

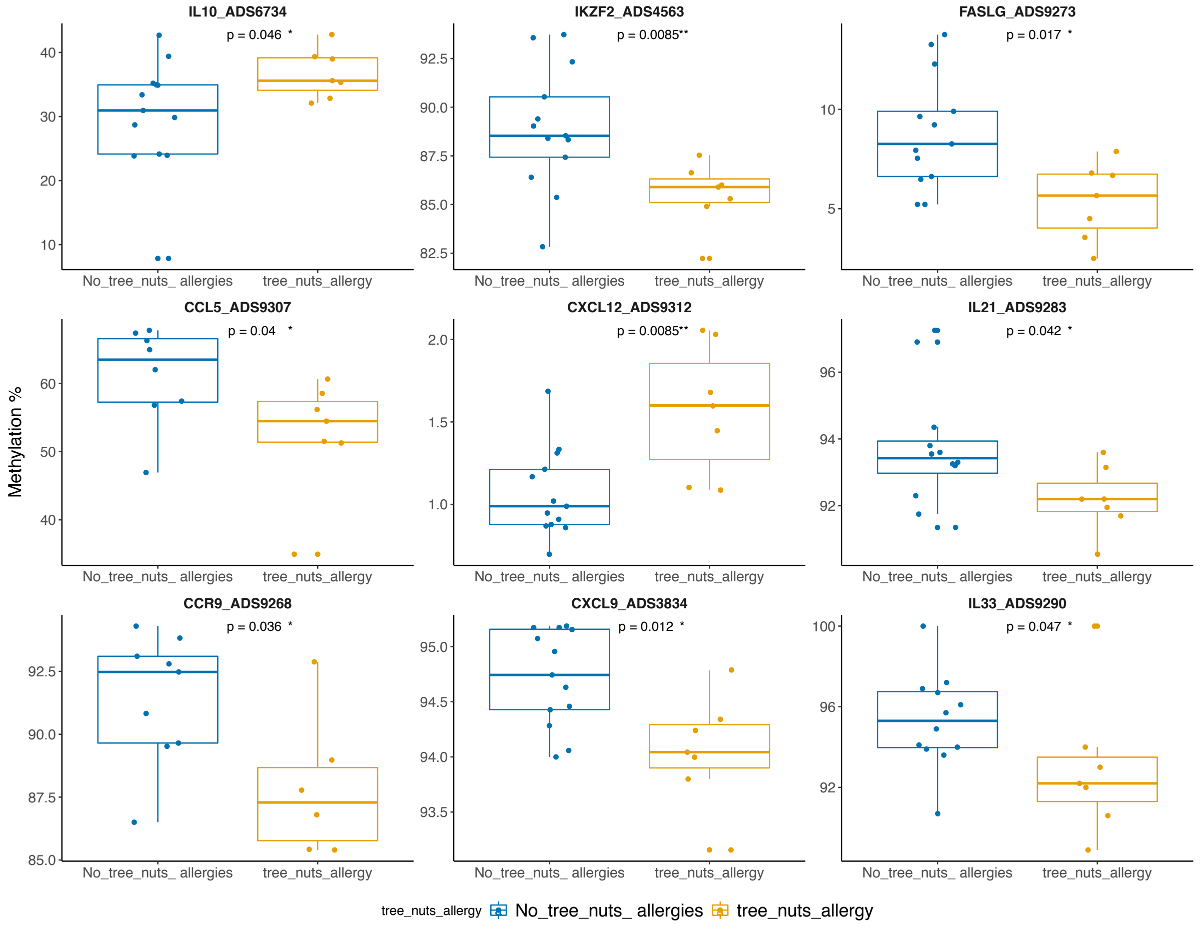

Supplementary Figure 4. The significant differences in the DNAm levels for targeted 9 genomic regions were observed between no tree nut allergy (n=13) and tree nut allergy (n=7) participants. The comparison analysis on the average DNAm level in each of 125 targeted genomic regions between no tree nut allergy (n=13) and tree nut allergy (n=7) participants was performed using nonparametric unpaired comparison test (Wilcoxon rank sum test). The box plots overlaid with dot plots showed the significant differences in the average methylation levels for 9 targeted genomic regions between no tree nut allergy (n=13) and tree nut allergy (n=7) participants (*p<0.05, **p<0.01)
